# A deep learning-based approach for rectus abdominis segmentation and distance measurement in ultrasonography

**DOI:** 10.3389/fphys.2023.1246994

**Published:** 2023-09-06

**Authors:** Fei Wang, Rongsong Mao, Laifa Yan, Shan Ling, Zhenyu Cai

**Affiliations:** ^1^ Center of Four-Dimensional Ultrasound, Affiliated Xiaoshan Hospital, Hangzhou Normal University, Hangzhou, Zhejiang, China; ^2^ Hangzhou Institute of Medicine, Chinese Academy of Sciences, Hangzhou, Zhejiang, China; ^3^ Department of Ultrasound, Zhejiang Medical and Health Group Hangzhou Hospital, Hangzhou, Zhejiang, China

**Keywords:** deep learning, diastasis recti abdominis, ultrasound, segmentation, rectus abdominis distance

## Abstract

**Introduction:** Diastasis recti abdominis (DRA) is a common condition in *postpartum* women. Measuring the distance between separated rectus abdominis (RA) in ultrasound images is a reliable method for the diagnosis of this disease. In clinical practice, the RA distance in multiple ultrasound images of a patient is measured by experienced sonographers, which is time-consuming, labor-intensive, and highly dependent on experience of operators. Therefore, an objective and fully automatic technique is highly desired to improve the DRA diagnostic efficiency. This study aimed to demonstrate the deep learning-based methods on the performance of RA segmentation and distance measurement in ultrasound images.

**Methods:** A total of 675 RA ultrasound images were collected from 94 *postpartum* women, and were split into training (448 images), validation (86 images), and test (141 images) datasets. Three segmentation models including U-Net, UNet++ and Res-UNet were evaluated on their performance of RA segmentation and distance measurement.

**Results:** Res-UNet model outperformed the other two models with the highest Dice score (85.93% ± 0.26%), the highest MIoU score (76.00% ± 0.39%) and the lowest Hausdorff distance (21.80 ± 0.76 mm). The average physical distance between RAs measured from the segmentation masks generated by Res-UNet and that measured by experienced sonographers was only 3.44 ± 0.16 mm. In addition, these two measurements were highly correlated with each other (*r* = 0.944), with no systematic difference.

**Conclusion:** Deep learning model Res-UNet has good reliability in RA segmentation and distance measurement in ultrasound images, with great potential in the clinical diagnosis of DRA.

## 1 Introduction

**FIGURE 1:**
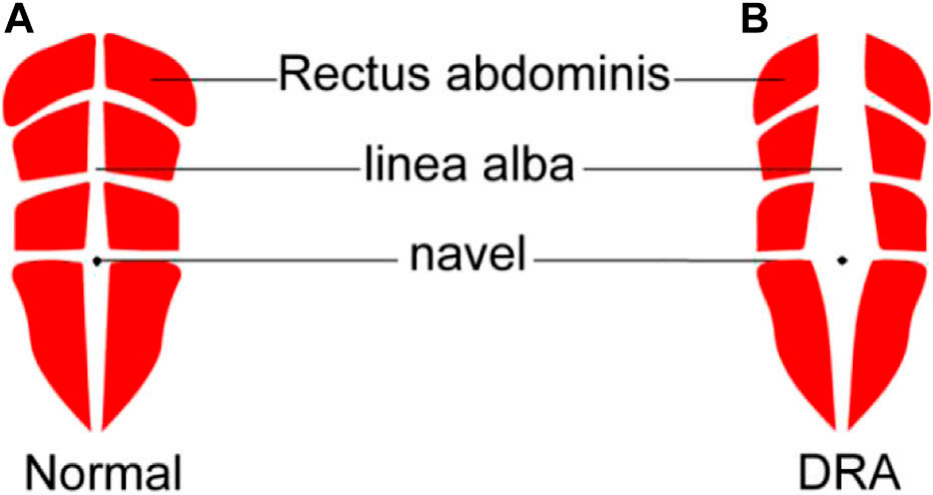
Schematic diagram of normal rectus abdominis **(A)** and DRA around navel **(B)**. DRA, diastasis recti abdominis.

Rectus abdominis (RA) is a long muscle located on both sides of the midline of anterior abdominal wall in rectus sheath. It plays an important role in protecting the internal organs and stabilizing the pelvis and lumbar spine. Diastasis recti abdominis (DRA) refers to a condition in which the two RAs separate to the sides, accompanied by extension of linea alba and protrusion of abdominal wall (Michalska et al., 2018) (Figure 1). The causes of DRA include changes in hormone levels, mechanical pressure, and other high-risk factors such as obesity, multiple pregnancies, multiparity, fetal macrosomia, polyhydramnios, and pre-pregnancy abdominal wall laxity (Bursch, 1987). After delivery, 20%–60% women experience varying degrees of DRA (Sperstad et al., 2016). This disease not only affects the abdominal aesthetics, but also causes other physical conditions such as the pain in lower back or knee (Fuentes Aparicio et al., 2021; Wu et al., 2021), leading to both psychological and physiological stress on patients. Therefore, the accurate diagnosis of DRA is meaningful to help patients to receive early rehabilitation trainings or surgical interventions (Fiori et al., 2021; Ross and Nahabedian, 2021), thus reducing the harm caused by DRA.

**FIGURE 2:**
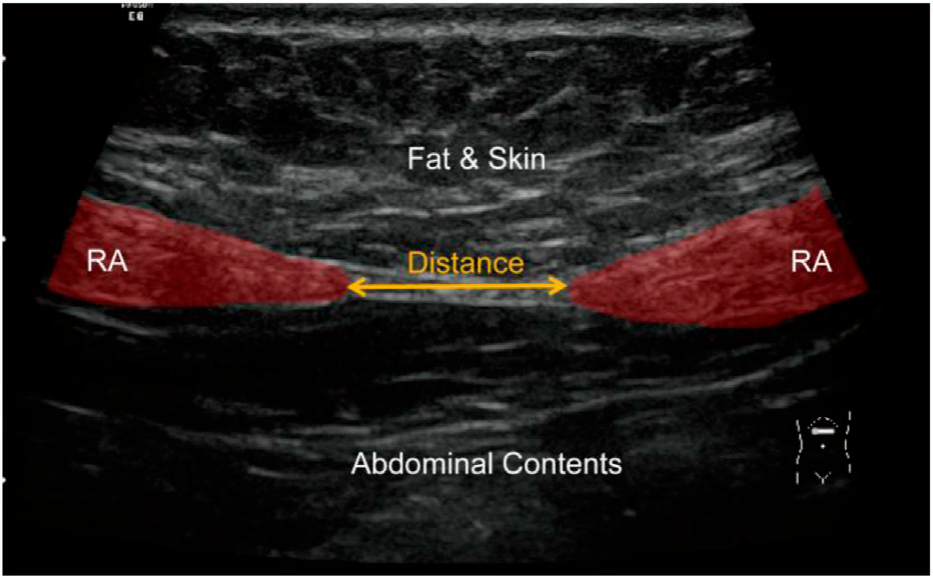
An ultrasound image showing RA (red regions) and measurement of RA distance (yellow arrow). RA, rectus abdominis.

In clinical practice, the diagnosis of DRA in *postpartum* women is performed by measuring the distance between RAs. This measurement can be done by several approaches including palpation (van de Water and Benjamin, 2016), ultrasound imaging (Mendes et al., 2007; Mota et al., 2012), and magnetic resonance imaging (Barbosa et al., 2013). Among these approaches, ultrasound imaging is the most widely used because it is non-invasive, real-time, and cost-effective. Figure 2 shows a representative ultrasound image of RA above the navel of a patient. The distance between two RAs (red regions) is measured to diagnose the degree of DRA. Usually, DRA is confirmed when the measured distance is larger than 2.5 cm. Otherwise, non-separation is reported. Many studies have demonstrated the reliability and effectiveness of ultrasound imaging in quantifying the separation between RAs for the diagnosis of DRA (Liaw et al., 2011; Mota et al., 2012; Qu et al., 2021). However, the RA distance is usually measured by sonographers with a manual method. This manual measurement is quite challenging for less-experienced sonographers because RA is hardly differentiated from its surrounding tissues in ultrasound images (Figure 2). In addition, for every patient, multiple (from 4 to 12) images obtaining from different locations in different posture states should be annotated, so the manual method is labor-intensive. Therefore, an automated method that can efficiently measure the distance between RAs in ultrasound images is highly desired.

To measure the distance between separated RAs in ultrasound images, the boundaries of two RAs should be first extracted. However, RA segmentation from ultrasound images is difficult because of the following reasons: 1) the boundary between RA and the surrounding tissue is weak; 2) the gray level and texture feature in RA are inhomogeneous and complicated; 3) the shape of RA varies across different images. Given all these difficulties, it is challenging for traditional segmentation techniques to achieve accurate RA segmentation in ultrasound images.

In recent years, deep learning methods have developed rapidly and been validated for their effectiveness in many medical fields (Akkus et al., 2019; Esteva et al., 2019; Kuo et al., 2019; Chan et al., 2020; Qian et al., 2021). Compared with traditional segmentation approaches, deep learning-based segmentation models, such as U-Net (Ronneberger et al., 2015) and their variants (Diakogiannis et al., 2020; Zhou et al., 2020), have achieved remarkable success with improvement in both time and accuracy. In this study, performances of three deep learning models including U-Net (Ronneberger et al., 2015), U-Net++ (Zhou et al., 2020) and Res-UNet (Diakogiannis et al., 2020) on the segmentation of RAs in ultrasound images were compared. Then, the distance between predicted RA masks was measured for diagnosis of DRA.

## 2 Materials and methods

### 2.1 Dataset collection

Dataset used in this study was collected from Affiliated Xiaoshan Hospital, Hangzhou Normal University (Hangzhou, China). In total, 675 ultrasound images of RA area obtained from 94 patients (aged 29.44 ± 3.98 years) at 42 days *postpartum* were obtained. For each patient, 4 to 11 ultrasound images were captured, including images in three posture states at four examination locations of RA during the examination. Specifically, three posture states referred to the relaxed state, curled-up state and deep inhalation state while retracting the abdomen towards the spine. Four examination locations referred to the RA area which was at 5 cm above navel, 3 cm above navel, navel itself and 3 cm below navel. The examination was performed using GE Voluson E8 ultrasound machine, with a high-frequency linear array transducer and depth range of 2–40 mm. The distribution of patients with different DRA conditions in ultrasound images was shown in Table 1.

**TABLE 1:** Distribution of patients with different DRA conditions in ultrasound images.

DRA condition	Patients number	Image number
Total	94	675
Separation only in resting state (≥2.5 cm)	27	190
Separation in all posture states (≥2.5 cm)	46	339
Non-diagnostic separation (<2.5 cm)	21	146

DRA, diastasis recti abdominis.

Each ultrasound image in the dataset had a size of 1,136 × 852 (pixels). The original images exported directly from the machine were in DICOM format. Three experienced sonographers in Affiliated Xiaoshan Hospital of Hangzhou Normal University were asked to manually label the RA regions from these ultrasound images. To evaluate the inter-annotator consistency, we calculated the Dice scores between the labeled masks of the three annotators We also calculated the Pearson correlation coefficients between the distance values measured by the three annotators. The high Dice score and correlation coefficient values shown in Table 2 indicate inter-annotator agreement. Finally, labels of the three annotators were averaged as the ground truth of the model. The manual annotations were checked for multiple times to ensure their reliability for being the ground truth masks while training a deep learning model. This study followed the tenets of Declaration of Helsinki, and was approved by the Ethics Committee of Affiliated Xiaoshan Hospital of Hangzhou Normal University. The informed consent was obtained from all patients.

**TABLE 2:** Consistency between different annotators.

	Dice (%)	Correlation coefficient (%)
Annotator 1–2	94.41	99.17
Annotator 2–3	94.66	99.36
Annotator 1–3	94.54	98.98

### 2.2 Deep learning-based segmentation of RAs in ultrasound images

**FIGURE 3:**
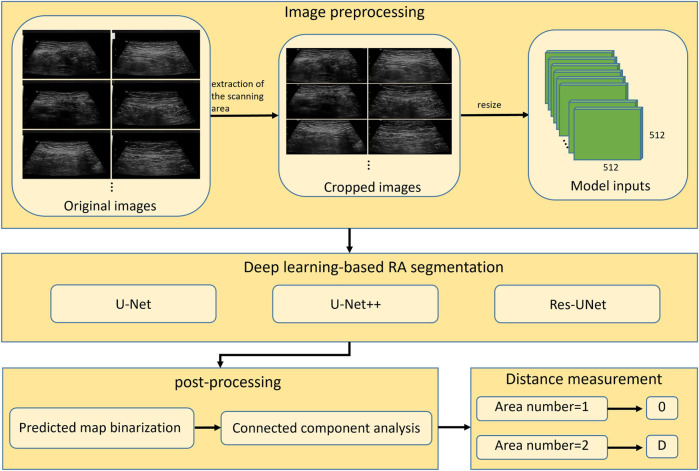
Overall workflow of automatic segmentation and distance measurement of RAs based on deep learning. RA, rectus abdominis; D, physical distance.

To measure the distance between two RAs in ultrasound images, the boundaries of two muscles should be accurately annotated, which is challenging for a non-experienced sonographer. In this study, we proposed to use deep learning-based methods to automatically segment RAs in ultrasound images. The overall workflow of proposed framework was shown in Figure 3. The method consisted of three steps including image preprocessing, deep learning-based RA segmentation, and mask post-processing.

#### 2.2.1 Image preprocessing

Since original ultrasound images are in DICOM format and have different sizes, they need to be preprocessed before feeding into a deep neural network. Firstly, we converted the original DICOM format files into PNG format images. Secondly, we removed the black regions those contained texts describing the information of ultrasound device and acquisition setup. These black regions were generally located in the periphery of image, around the ultrasound scanning area. We binarized the image using Otsu’s thresholding technique (Otsu, 1979), and then performed the connected component analysis to obtain the largest connected region. We cropped the minimum enclosing rectangle of this region from the original image, and thus removed the periphery black regions. Finally, the cropped images were resized to 512 × 512 (pixels) to feed into deep-learning segmentation models.

#### 2.2.2 Deep learning-based RA segmentation

We demonstrated three models including U-Net (Ronneberger et al., 2015), UNet++ (Zhou et al., 2020), and Res-UNet (Diakogiannis et al., 2020) on ultrasound RA segmentation. U-Net, as one of the most classic segmentation model, is widely used in the medical field and serves as a baseline for many other networks. UNet++ improves upon U-Net by changing the network’s connectivity, making it more suitable for medical image segmentation. Res-UNet replaces the sub-modules in U-Net with residual blocks from ResNet (He et al., 2016), effectively improving the segmentation performance. These three models have been widely applied and shown good performance in various segmentation tasks. By utilizing these three classic deep learning models, we can fully validate the feasibility of deep learning methods for RA segmentation task.

Preprocessed images obtained by methods described in Section 2.2.1, were split into training (63 patients, 448 images), validation (12 patients, 86 images), and test sets (19 patients, 141 images) based on a ratio of 7: 1: 2. Because there may be information redundancy between different images of the same patient, the dataset was divided at the patient level, so that different images of the same patient would not appear in two subsets simultaneously. Data augmentation including horizontal flipping and contrast change was used to increase the diversity of dataset and avoid the risk of overfitting.

To optimize the model parameters, we used the cross-entropy loss as the loss function. The cross-entropy loss measures the difference between predicted segmentation and ground truth mask, which is defined as:

$$L\left(x,y\right)=-\sum y\log x$$

where x is the predicted segmentation result, and y is the annotated ground truth.

**FIGURE 4:**
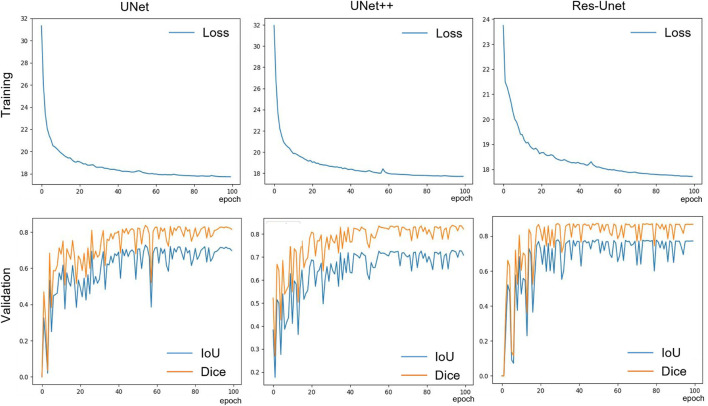
The training loss and validation set IoU and Dice curves of the three models.

All models were implemented in Pytorch 1.12.1 framework and trained on a single 3090 GPU for 100 epochs with an initial learning rate of 0.001 and a momentum value of 0.9. During training, Adaptive Moment Estimation (Adam) (Kingma and Ba, 2015) was used to optimize the training process. The batch size was set to 8 to accommodate the size of our GPU memory. The model updated its parameters by learning the effective information in the images through forward and backward propagation. The validation was performed using the validation set to determine the convergence of the model. After training, we selected the model parameters with the best performance on the validation set for testing. The saved model parameters were used to evaluate the model’s segmentation and generalization performance on the test set. During the validation and testing phases, the backpropagation of the model was halted, so that the model did not update its parameters. The training curves of the three models (Figure 4) indicate that the model gradually fitted the dataset during the training process. Based on this curve, we developed an early stop strategy to solve the overfitting problem of the model.

#### 2.2.3 Mask post-processing

**FIGURE 5:**
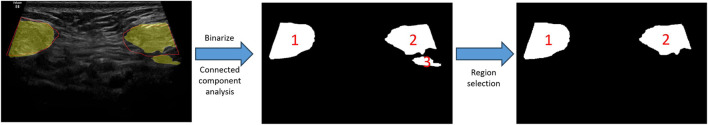
Schematic diagram of post-processing.

Outputs of the trained models are maps showing the probability of pixels belonging to RAs. The predicted probability maps were binarized by a threshold value of 0.5 to create segmentation masks. Since the separated RAs usually appear as two disconnected regions in ultrasound images, there will be segmentation errors if the number of segmented regions is greater than two (as shown in Figure 5). This type of error was corrected by performing connected component analysis on the binary mask image, and the two largest connected components were selected as the final RA regions.

### 2.3 RA distance measurement

**FIGURE 6:**
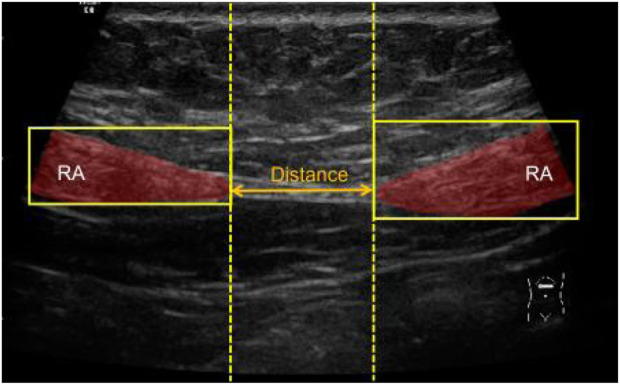
Schematic diagram of measuring the distance between two segmented RAs. RA, rectus abdominis.

After mask post-processing, there were one or two connected regions in the final binary mask. If there was only one connected region in the segmentation mask image, the RA was thought to be non-separated, and the RA distance was set to 0. If there were two connected regions in the segmentation mask image, the distance between two regions was calculated as the RA separation distance. Specifically, the minimum bounding rectangles of two regions were extracted, and then the distance between right edge of left rectangle and left edge of right rectangle was calculated as the final RA distance (*d*) in pixels (Figure 6). Then, the physical distance (D) between two RAs was computed by the following equation:

$$D=P_{x}*d$$

where $P_{x}$ represents the pixel size of the image after the resize operation.

### 2.4 Evaluation

Three metrics including Dice coefficient, mean intersection-over-union (MIoU) and Hausdorff distance (HD) were calculated for evaluating the segmentation performance of three deep learning models. The equations for computing Dice, MIoU and HD were as follows:

$$Dice=\frac{2\left|A\cap B\right|}{\left|A\right|+\left|B\right|}$$

$$MIoU=\frac{\left|A\cap B\right|}{\left|A\cup B\right|}$$

$$HD=\max\left(h\left(A,B\right),h\left(B,A\right)\right)$$

where A was the model prediction mask, B was the ground truth mask, $h\left(A,B\right)$ represented the maximum of the shortest distance from each pixel in A to B, and $h\left(B,A\right)$ represented the maximum of the shortest distance from each pixel in B to A . The larger the Dice and MIoU and the smaller the HD, the better the segmentation.

We defined a metric average physical distance error (APDE) to compare the RA distance calculated by our method with manual measured distance. APDE was expressed as:

$$APDE=\frac{1}{N}\sum_{i}^{N}p^{\left(i\right)}\left|d^{\left(i\right)}-{\hat{d}}^{\left(i\right)}\right|$$

where N was the number of image samples, $d^{\left(i\right)}$ was the RA distance in image sample *i* manually measured by experienced sonographers, ${\hat{d}}^{\left(i\right)}$ was the computed distance between the two largest connected domains predicted by our model in image sample *i*, and $p^{\left(i\right)}$ was the pixel size of image sample *i*.

## 3 Results

### 3.1 RA segmentation results of three deep learning models

**FIGURE 7:**
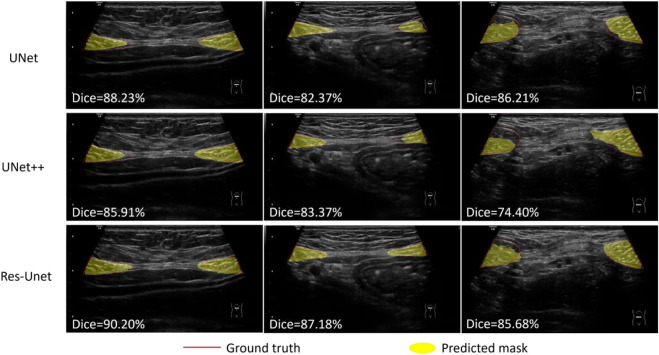
Segmentation results using three models on three rectus abdominis ultrasound images.

**FIGURE 8:**
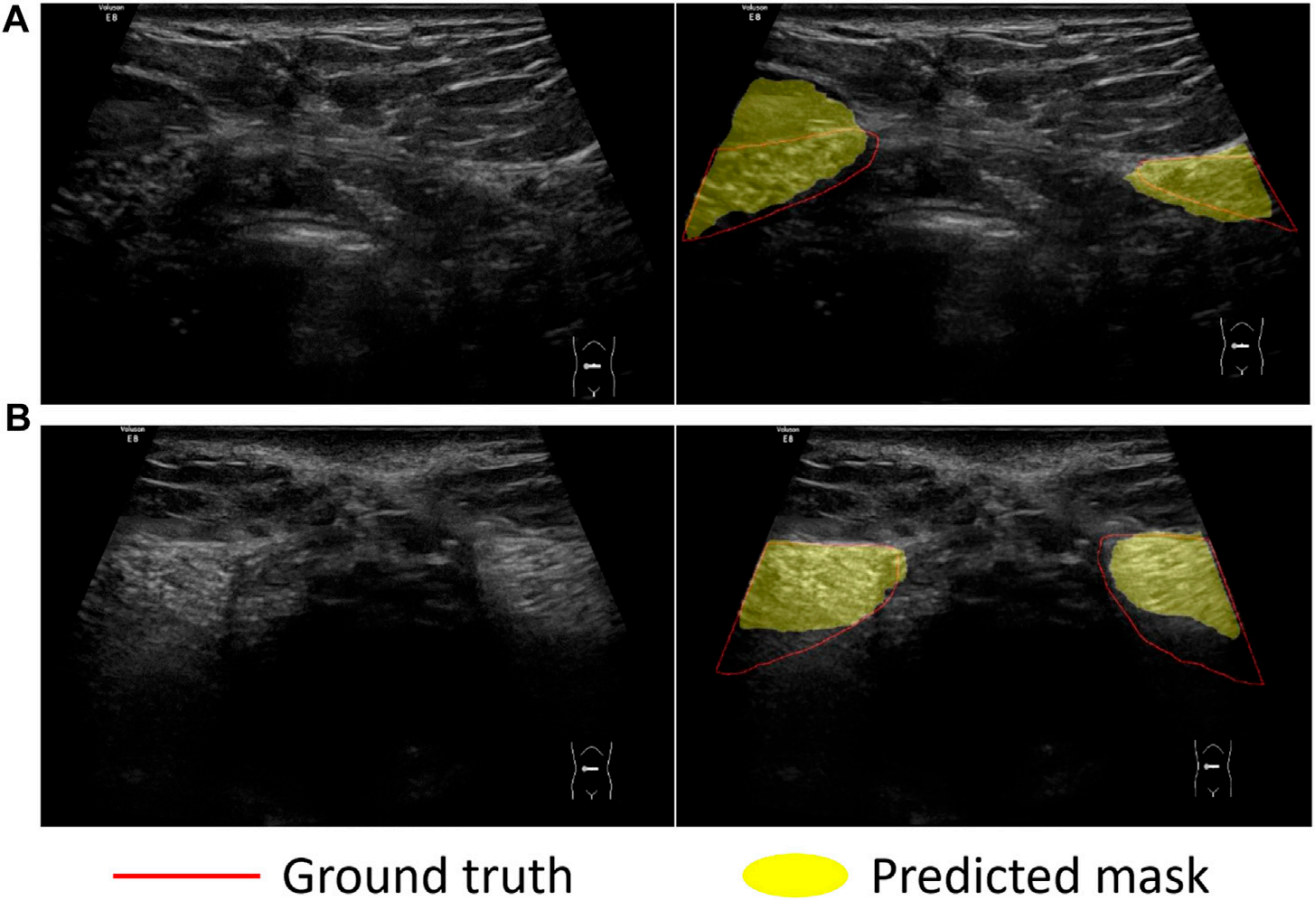
Sample examples that cannot be easily segmented. Challenges in dividing examples: **(A)** Complex organizational structure; **(B)** Weak edges.

We compared the performance of three models including U-Net, UNet++ and Res-Unet on RA segmentation in ultrasound images. After five independent repeated experiments, the evaluation metrics calculated on the test dataset using three models were shown in Table 3. Res-Unet outperformed U-Net and UNet++ in all four metrics with the largest Dice score (85.93% ± 0.26%), the largest MIoU score (76.00% ± 0.39%), the smallest HD score (21.80 ± 0.76 mm) and the smallest APDE (3.42 ± 0.16 mm). The segmentation results of three exemplar ultrasound images by the three models were shown in Figure 7. We observed that a subset of samples exhibited suboptimal segmentation results, as shown in Figure 8. The complex organizational structure leads to imperfect segmentation performance in Figure 8A, while weak edges lead to unsatisfactory segmentation performance in Figure 8B.

**TABLE 3:** Performance of three models on RA segmentation and distance measurement.

Method	Dice (%)	MIoU (%)	HD (mm)	APDE (mm)
U-Net	81.21 ± 0.93	69.66 ± 1.21	28.75 ± 3.15	4.72 ± 0.42
U-Net++	82.31 ± 1.06	71.28 ± 1.26	28.18 ± 4.90	4.62 ± 0.84
Res-UNet	85.93 ± 0.26	76.00 ± 0.39	21.80 ± 0.76	3.44 ± 0.16

RA, rectus abdominis; MIoU, mean intersection-over-union; HD, hausdorff distance; APDE, average physical distance error. The Res-UNet, method is significantly superior to the other two methods (*p* <0.05).

### 3.2 RA distance measurement

**FIGURE 9:**
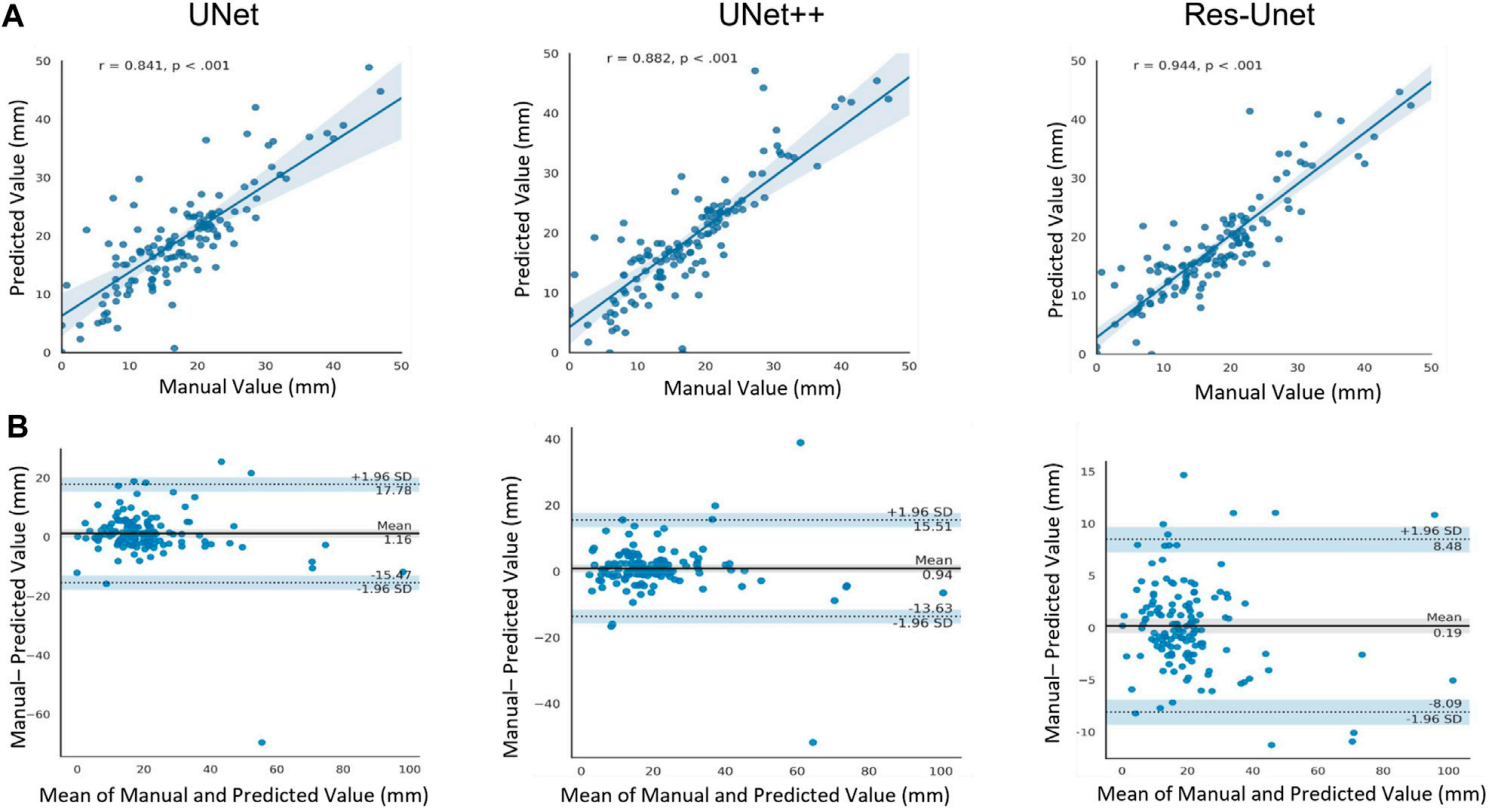
**(A)** Scatter plot of Pearson correlation analysis between computed values and manually measured values (ground truth). r, Pearson correlation coefficient. **(B)** Bland-Atman plot between the predicted rectus abdominis distance and manually measured values.

We compared the RA distance of 141 test images between computed using the three model and measured manually by experienced sonographers. Figure 9A showed the scatter plot of RA distance computed from the proposed method (*y*-axis) versus manual ground truth values (*x*-axis). The Pearson correlation coefficient of the Res-UNet method was calculated to be 0.944 with *p* < 0.001. This indicated a strong positive correlation between the measured and true values and a high degree of statistical significance. Figure 9B showed the Bland-Altman analysis of two measurements for three methods. The line of equality (Predicted - Manual = 0) was within the 95% confidence interval of mean difference, illustrating no significant systematic difference between two measurements.

## 4 Discussion

DRA is a common condition in *postpartum* women, and it is harmful to the physical and mental health of patients if left untreated (Fuentes Aparicio et al., 2021; Wu et al., 2021). Manually measuring the distance between separated RAs in ultrasound image has been a golden standard for the diagnosis of DRA (Keshwani et al., 2018). However, due to the measurement at multiple locations and in multiple posture states of patient, the manual method can be time-consuming and labor-intensive. Additionally, the recognition of RAs from an ultrasound image is quite challenging for an inexperienced sonographer since RAs are very similar to their surrounding tissues. Therefore, an automatic method that can efficiently segment RAs and measure RA distance in ultrasound images would benefit an objective and accurate diagnosis of DRA.

In this study, we proposed a fully automatic pipeline based on deep learning methods for RA segmentation and measurement of RA distance. The most commonly used deep learning segmentation model U-Net, and its two variants U-Net++ and Res-UNet, were evaluated on the ultrasound segmentation of RAs. Results showed that, Res-UNet model outperformed the other two models with the highest Dice score (85.93% ± 0.26%), the highest MIoU score (76.00% ± 0.39%) and the lowest Hausdorff distance (21.80 ± 0.76 mm). The average physical distance of RAs between measured by Res-UNet model and measured by experienced sonographers was only 3.44 ± 0.16 mm. In addition, these two measurements were highly correlated with each other (*r* = 0.944), with no systematic difference.

Our study has provided significant insights into the effectiveness of proposed methods for the segmentation and measurement of RA distance in ultrasound images. Firstly, the high correlation between our automatic RA distance measurements and the ground truth manual measurements by sonographers indicates the validity and reliability of our methods. The Bland-Altman analysis further supports this finding, showing no significant systematic difference between the two measurements. This suggests that our automatic measurements can serve as a reliable alternative to manual measurements, saving time and effort for clinicians. Secondly, our segmentation models, particularly Res-UNet, demonstrate excellent performance in segmenting the RA region, as evidenced by the high Dice coefficient, high MIoU, and small HD. This indicates that our deep learning-based approach effectively captures RA boundaries and accurately separates it from surrounding tissues, which is crucial for precise distance measurements. Furthermore, the small measurement errors achieved by our method in the task of RA separation measurement highlight its accuracy in quantifying the degree of separation. This is crucial for diagnosing DRA and monitoring its progression. The combination of accurate segmentation and precise distance measurement enhances the diagnostic capability of our method, providing valuable information for healthcare professionals.

Although the methods used in our study have been demonstrated to measure RA distance reliably and accurately in ultrasound images, this study still has certain limitations. Due to the difficulty of data collection and annotation, and ethical issues, the amount of data is relatively small, with only 93 patients having been investigated. Therefore, a larger dataset is needed to evaluate the effectiveness of our workflow. Nevertheless, we believe that our experiment can still prove the potential of deep learning algorithms in DRA assessment and can be applied to the future screening of DRA.

Our study has demonstrated the effectiveness of deep learning-based methods in the segmentation and measurement of RA and diagnosis of DRA. The integration of advanced imaging technology with deep learning algorithms has the potential to revolutionize the assessment and screening of DRA, providing objective and accurate measurements that benefit both healthcare professionals and patients. Further research and clinical validation are warranted to fully explore the clinical utility and applicability of our proposed methods in the field of DRA assessment.

## 5 Conclusion

In this study, we demonstrated the ability of deep learning-based methods on RA segmentation in ultrasound images. The method we proposed to measure RA distance correlate very well with manual ground truth, thus can be reliably used for evaluating RA separation degree and has great potential to improve the clinical workflow of DRA diagnosis.

## Data Availability

The original contributions presented in the study are included in the article/Supplementary Materials, further inquiries can be directed to the corresponding author.
